# Transforming Rapid Diagnostic Tests for Precision Public Health: Open Guidelines for Manufacturers and Users

**DOI:** 10.2196/26800

**Published:** 2022-07-29

**Authors:** Peter Lubell-Doughtie, Shiven Bhatt, Roger Wong, Anuraj H Shankar

**Affiliations:** 1 Ona Systems Inc Burlington, VT United States; 2 Centre for Tropical Medicine and Global Health Nuffield Department of Medicine University of Oxford Oxford United Kingdom; 3 Eijkman-Oxford Clinical Research Unit Jakarta Indonesia

**Keywords:** rapid diagnostic test, precision public health, digital health, diagnostic, testing, guideline, manufacture, surveillance, FHIR, Fast Healthcare Interoperability Resources

## Abstract

**Background:**

Precision public health (PPH) can maximize impact by targeting surveillance and interventions by temporal, spatial, and epidemiological characteristics. Although rapid diagnostic tests (RDTs) have enabled ubiquitous point-of-care testing in low-resource settings, their impact has been less than anticipated, owing in part to lack of features to streamline data capture and analysis.

**Objective:**

We aimed to transform the RDT into a tool for PPH by defining information and data axioms and an information utilization index (IUI); identifying design features to maximize the IUI; and producing open guidelines (OGs) for modular RDT features that enable links with digital health tools to create an RDT-OG system.

**Methods:**

We reviewed published papers and conducted a survey with experts or users of RDTs in the sectors of technology, manufacturing, and deployment to define features and axioms for information utilization. We developed an IUI, ranging from 0% to 100%, and calculated this index for 33 World Health Organization–prequalified RDTs. RDT-OG specifications were developed to maximize the IUI; the feasibility and specifications were assessed through developing malaria and COVID-19 RDTs based on OGs for use in Kenya and Indonesia.

**Results:**

The survey respondents (n=33) included 16 researchers, 7 technologists, 3 manufacturers, 2 doctors or nurses, and 5 other users. They were most concerned about the proper use of RDTs (30/33, 91%), their interpretation (28/33, 85%), and reliability (26/33, 79%), and were confident that smartphone-based RDT readers could address some reliability concerns (28/33, 85%), and that readers were more important for complex or multiplex RDTs (33/33, 100%). The IUI of prequalified RDTs ranged from 13% to 75% (median 33%). In contrast, the IUI for an RDT-OG prototype was 91%. The RDT open guideline system that was developed was shown to be feasible by (1) creating a reference RDT-OG prototype; (2) implementing its features and capabilities on a smartphone RDT reader, cloud information system, and Fast Healthcare Interoperability Resources; and (3) analyzing the potential public health impact of RDT-OG integration with laboratory, surveillance, and vital statistics systems.

**Conclusions:**

Policy makers and manufacturers can define, adopt, and synergize with RDT-OGs and digital health initiatives. The RDT-OG approach could enable real-time diagnostic and epidemiological monitoring with adaptive interventions to facilitate control or elimination of current and emerging diseases through PPH.

## Introduction

### Background

Rapid diagnostic tests (RDTs), specifically immunochromatographic lateral flow assays, can provide accurate real-time point-of-care diagnoses in low-resource settings and have been an important tool in the global health arsenal. Advances in microfluidics have enabled the medical device community to design smaller RDTs—as small as half the area of a business card and the thickness of a watch—that can be used to diagnose more conditions and are low cost—less than US $1 per device [1]. Manufacturers and international health organizations have collaborated to deliver hundreds of millions of RDTs to countries and communities [2]. However, the full potential of RDTs as a public health tool has not yet been realized. This is due to field deployment challenges amplified by a fragmented market, nonstandard designs, and lack of features that facilitate systematic capture and use of RDT results and patient data. Fortunately, current technology—computer vision, widely deployed smartphones, and mobile networks—applied to RDTs provides a scalable path to improved health and well-being through the emerging paradigm of precision public health (PPH). PPH is a field that aims to maximize impact with the active use of data for surveillance and targeted interventions by temporal, spatial, and epidemiological characteristics of populations.

Therefore, we aimed to define axioms to underpin steps to incorporate RDTs into a PPH approach, identify an initial set of features (RDT’s hardware and software features) that would be needed to implement these axioms, and select a final evidence-based set of features that could be used by health policy and program implementers in integrating RDTs as tools for frontline health care workers at the community and clinic levels [3,4].

### Challenges Facing the Current RDT Ecosystem

There are 3 core challenges faced within the current RDT ecosystem, which impede application and widespread implementation for PPH.

#### Lack of Data Standards

**Figure 1:**
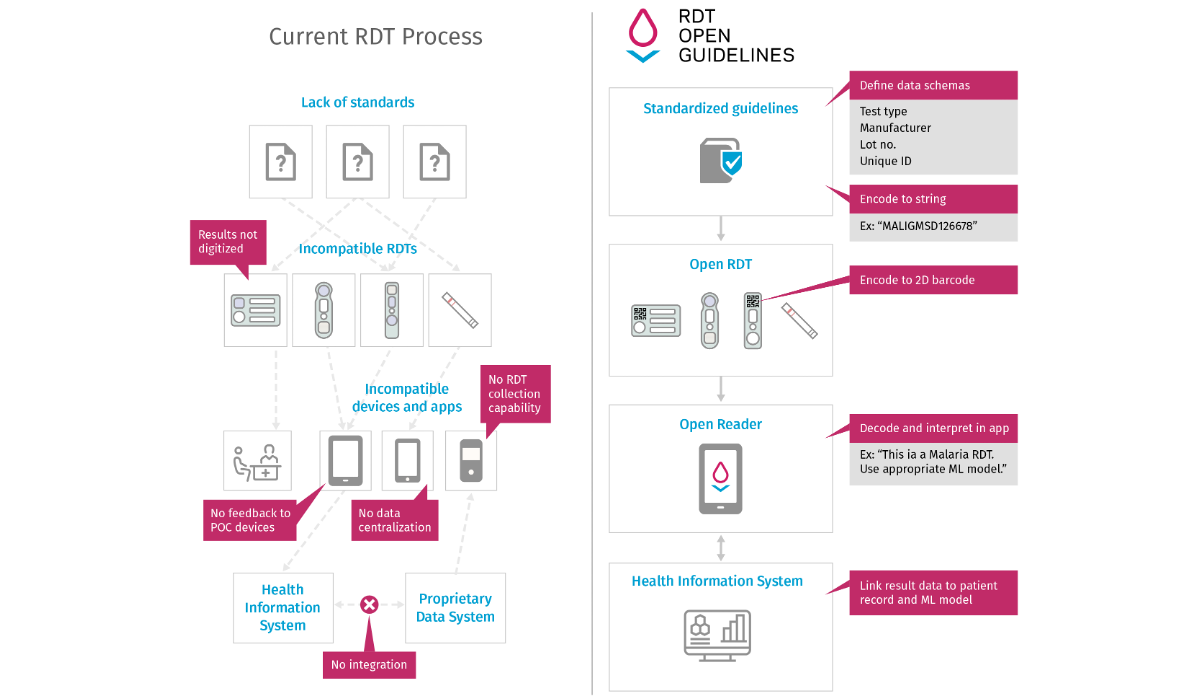
Current rapid diagnostic test processes (left) use undefined or proprietary standards, which lead to multiple incompatible protocols (ie, incompatible apps and devices). The rapid diagnostic test open guideline process (right) uses standard guidelines to produce modular reference rapid diagnostic tests that are compatible and can be read by a well-defined protocol for devices and apps. ID: identifier code or number for each device; ML: machine learning; POC: point-of-care; RDT: rapid diagnostic test.

The lack of uniformity in RDT hardware and software substantially limits the integration of public health data from RDTs into current health information systems, thereby impeding their ability to respond to emerging crises [5]. Under these conditions, bridging these limitations requires analytics-intensive tasks to convert, code, recode, and integrate data. Current RDT versions require specific knowledge and tools that are typically not compatible (Figure 1), leading to a combinatorial explosion of integration and data interoperability requirements. Without uniformity, health professionals and individual consumers using RDTs cannot benefit from integration with point-of-care smartphone apps to enable personal tailored care guided by modern machine learning techniques that can calculate the prior probabilities of having a condition from data on demography, the environment, and etiology.

#### Heterogeneity of RDT Reader Hardware

One strategy to improve the uniformity, amount, and quality of information collected from RDTs is to use custom hardware readers and image capture and analysis devices (eg, the DekiReader [6], specialized microscopes, and device holders). However, these devices can be problematic owing to the expense, continuous supply, and maintenance required. As such, custom hardware is incompatible with large-scale deployments and the broad consumer use necessary for RDTs to cover high- and emerging-risk areas; therefore, such devices hinder the continuous stream of accurate diagnostic data needed to expose outbreaks of known diseases and predict the emergence of new diseases.

#### Diversity of RDT Form Factors and Instructions

Any diagnostics integrated with smartphones would still involve manual use of an RDT and interaction with patients. However, several studies have documented the challenges faced by health care workers in translating their competency to use one RDT to comparable competency with another (ie, those for similar diseases, from other manufacturers, or with revised procedures). The lack of consistency contributes to high error rates in RDT usage and interpretation and limits their impact [7].

### Solving These Challenges With Open Guidelines

Based on these challenges, we define 3 axioms that underpin solutions (Table 1). To solve the challenges and maximize information usage for PPH, RDTs should adhere to a set of open guidelines (OGs), and use smartphone readers and data protocols that standardize the information—both horizontally between different manufacturers or providers and vertically between different steps in the RDT life cycle (Figure 1). These axioms can transform the current state of the RDT ecosystem into one that supports PPH. RDT-OGs address the data uniformity challenge of RDTs by standardizing the capture and use of data.

**Table 1:** Rapid diagnostic test open guideline axioms.

Axiom	Description
Axiom 1: Maximize rapid diagnostic test (RDT) data usage by capturing and structuring information for integration	To address the custom hardware challenge, we designed RDT open guidelines (OGs) in line with the existing realities of the rapid diagnostic test manufacturing world. Manufacturers focused on creating tests simple enough to be used in clinical, community, and household settings by minimally trained community health workers, and eventually clients themselves. This need for widespread use leads us to define RDT-OGs to satisfy and facilitate these needs.
Axiom 2: Any solution must rely on only readily available local resources	This allows implementers of RDT-OGs to create solutions accessible by locally available technology and capability, including those that use pre-existing devices in target communities, such as low-cost smartphones [8]. RDT-OGs address the problems caused by lack of physical device uniformity by designing for human and device interoperability.
Axiom 3: Diagnostic interfaces should remain uniform or compatible	This applies to RDT hardware, the software reading and interpreting RDTs, and the data schemas integrating with external systems. When using software-based RDT readers, following Axiom 3 leads us to link individual RDTs to uniform interactive guidance of users as they conduct a diagnostic test.

### The Need to Catalyze the Era of RDT-OGs

**Figure 2:**
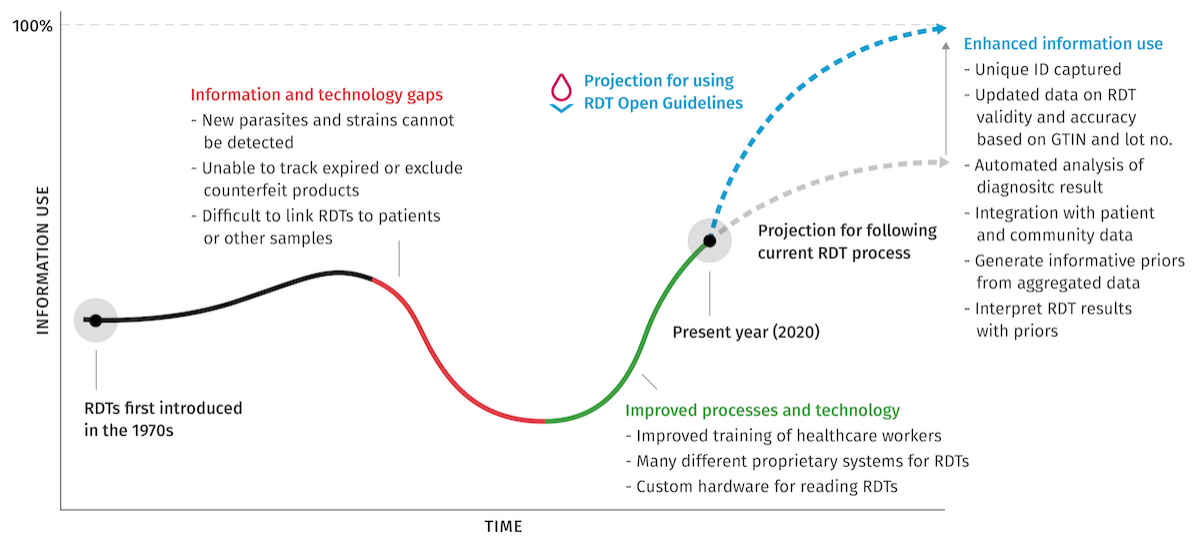
The trajectory of information use in response to rapid diagnostic test technology innovation, which shows the introduction of rapid diagnostic tests and their initial impact (black line) followed by subsequent challenges (red line) and improvements (green line). Potential future paths are also shown: a lower growth in information utilization under the current incremental improvements (gray dashed line) or an accelerating trajectory enabled with rapid diagnostic test open guidelines (blue dashed line). GTIN: global trade item number; RDT: rapid diagnostic test.

We have chosen to address the current challenges in RDTs with open guidelines in order to focus directly on the systems and integration problems they face. Figure 2 shows initial progress as RDTs were developed in the laboratory; as researchers predicted their impact, optimism surrounding their potential grew. As RDT rollouts began, the ability to capture diagnostic data increased, but these increases did not keep pace with global growth in the technological capacity to store, communicate, and analyze information [9].

This changing technology landscape, combined with a lack of individual RDT identifiers, inconsistent test use protocols, and the appearance of fraudulent and counterfeit RDTs, led to a relative decrease in information use; however, as the RDT community began to effectively encode and aggregate information and improve the training of health care workers, information use increased. Currently, RDTs have reached a tipping point—there are multiple proprietary hardware and software solutions, and medical systems are facing “information overload” [10]. The future trend in information use could take 1 of 2 diverging paths—modest growth with eventual stagnation or a promising future with open guidelines aligned with the aforementioned PPH axioms to accelerate impact by enhancing information usage (Figure 2).

Below, we describe our methods, present survey results from experts in RDT technology, formally establish an information utilization index (IUI), and define how various RDT features gather information. We use this to assess World Health Organization (WHO)–prequalified RDTs in comparison to a prototype RDT based on open guidelines and then discuss these results and related work in field-based hardware and software diagnostics and standards. As access to telecommunications networks improves worldwide, and advanced information systems become more widely used by ministries of health and global health organizations, the community can dramatically accelerate the transformational impact promised by RDTs.

## Methods

### Review of Published Literature

To identify key issues related to data capture and use from RDTs, we conducted a review of published papers using the Semantic Scholar artificial intelligence–enabled research search engine [11]. We focused on, but did not constrain ourselves to, PubMed-indexed medical journal papers. The search was conducted on December 31, 2018, and updated on December 31, 2019, using the keywords “rdt,” “smartphone,” and “mobile phone.” The search revealed 480 papers that were further screened for the study of lateral flow immunochromatographic rapid tests, yielding 58 papers. In addition to reviewing these papers, we reviewed their citations to identify additional papers in telecytology, immunochromatography, and diagnostic hardware. From these papers, we extracted themes and concepts related to barriers to information capture and usage from RDTs, and applied a grounded theory conceptual framework to compile and code themes and concepts into core ideas and then high-level abstractions and classifications. These were discussed and reviewed by 3 different members of the team. We considered this process complete when saturation was reached (ie, no additional novel ideas or abstractions emerged upon review of additional papers).

### RDT Stakeholder Survey

#### Procedure

The literature review and PPH axioms were used to identify the fundamental features and feasibility of open guidelines for RDTs that maximize information usage for PPH. A survey was designed, which comprised 30 questions (Multimedia Appendix 1). Respondent-driven sampling was used and initiated by contacting authors of the papers reviewed and professional referrals, which included researchers, medical technologists, manufacturers, medical professionals, and frontline health workers. These stakeholders were asked to participate in a web-based survey that comprised specific statements that corresponded to general, user-specific, manufacturer-based issues or issues regarding informatics. A 5-point Likert scale was used for response: 0=strongly disagree, 1=disagree, 2=neutral, 3=agree, 4=strongly agree, and unable to reply. Replies were accrued from January 2019 to March 2019, and submitted entries were downloaded and tabulated. Results were summarized by tabulations and analysis of proportions using Excel (version 16; Microsoft Inc).

#### Ethics Approval

We note that the survey was exempt from human subjects research as per guidelines from the US Department of Health and Human Services as it assessed a public benefit or service and was not about humans, and did not collect sensitive information.

### Defining the IUI

Survey results and the literature review were used to identify essential information features for RDTs and their integration into health care platforms to support PPH. The presence or absence of a feature for a specific RDT could be used to calculate an IUI defined as *number of features present* or the *number of features defined*. We then selected WHO prequalified cassette-based RDTs for malaria and HIV that had been assessed for performance [12] and calculated the IUI.

## Results

### Literature Review

The review of published literature and thematic extraction of concepts related to information capture and usage from RDTs led us to identify the following core areas that affect information usage (Table 2).

**Table 2:** Core areas that affect information usage.

Core areas	Description
Challenges in using commonly deployed rapid diagnostic tests (RDTs).	This is related to issues of RDT choreography, and proper reading and interpretation even when control and results lines were clear.
Existing barriers for mobile imaging of RDTs.	This referred to shadows from the cassette on the surface of the immunochromatographic strip, or glare from the cassette and surface of the strip, all of which hindered image capture quality.
Criteria for designing RDT standards.	This included specific characteristics of RDTs that could be feasibly standardized.
Barriers to RDT manufacturing standards.	This referred to cost and other factors that could hinder manufacturing to an enhanced standard.
Feasibility and features for smartphone-read RDTs.	This included the practicality of using identified features in the clinical or field setting.
Perceptions of non–human-readable RDTs (eg, electrochemical readouts).	This included whether or not read-out systems for RDTs would be acceptable for clinical or field personnel, if the actual reaction was not observable.

### RDT Stakeholder Survey

We contacted 81 stakeholders, and 33 completed the questionnaire (16 researchers, 7 technologists, 3 manufacturers, 2 doctors or nurses, and 5 others). Respondents were most concerned about the proper use of RDTs (agreed: 30/33, 91%), their interpretation (agreed: 28/33, 85%), and reliability (agreed: 26/33, 79%). Respondents were confident that smartphone-based RDT readers could address some reliability concerns (agreed: 28/33, 85%) and that readers were more important for complex or multiplex RDTs (agreed: 33/33, 100%).

### IUI

Based on these results, and because RDTs are embedded in a set of protocols and practices defined by health care workers, institutions, clients, and communities, proper usage of rapid diagnostic tests depends not only on a physical device but also on its integration into the larger ecosystem. In this context, to maximize information usage, an RDT platform must function effectively in all phases of its life cycle, with added value at each phase.

**Figure 3:**
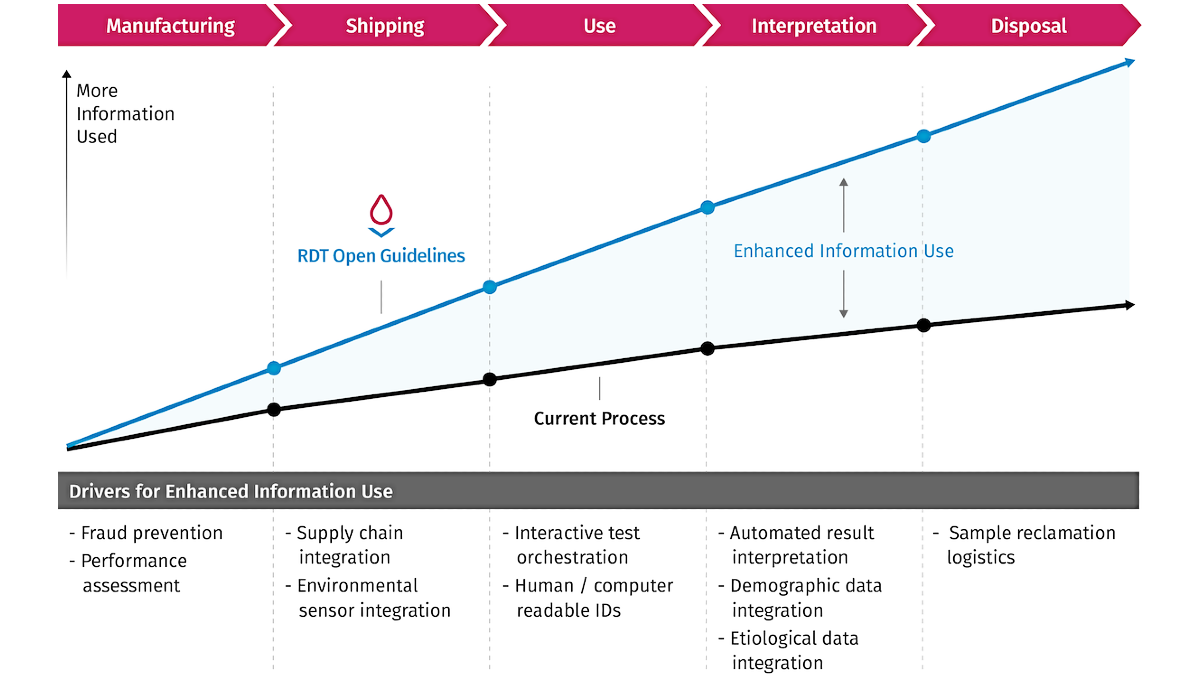
Conceptual framework of gaps in information use through the rapid diagnostic test life cycle. Information utilization (vertical axis) is quantified relative to 5 distinct phases of the rapid diagnostic test life cycle (horizontal axis): Manufacturing, Shipping, Use, Interpretation, and Disposal. Over the life cycle, the information utilization of the current process increases (black line), but with the use of rapid diagnostic test open guidelines, information utilization would increase (blue line) as a result of several features (list at bottom).

Specific stakeholder results are divided into discrete phases of the rapid diagnostic test life cycle (Manufacture, Shipping, Use, Interpretation, and Disposal), and RDT capabilities are divided into themes (Metadata, Molding of the Cassette, Printed Data, and Smartphone Reader) (Figure 3). The open guidelines contribute to each theme, and they are essential for the Smartphone Reader theme. A conceptual framework that contrasts the accumulated value of RDT open guidelines and the current RDT process shows an increase at each life cycle phase. Specific capabilities drive these increases (Figure 3).

We identified 11 essential information features for RDTs and their integration into health care platforms, which define components of the IUI (Textbox 1).

**Components of the information utilization index.**

1. Smartphone or other device reader exists
2. Instructions included
3. Cassette is not reflective
4. Test strip is not reflective
5. Shadow does not exist on test window
6. Expiration date printed on device
7. Identifier printed on device
8. Color calibration panel on device
9. 2D barcode on device
10. Test name clearly printed on device
11. Regulator (eg, World Health Organization) approved for lab and field use

### Assessment of Current Rapid Diagnostic Tests

**Figure 4:**
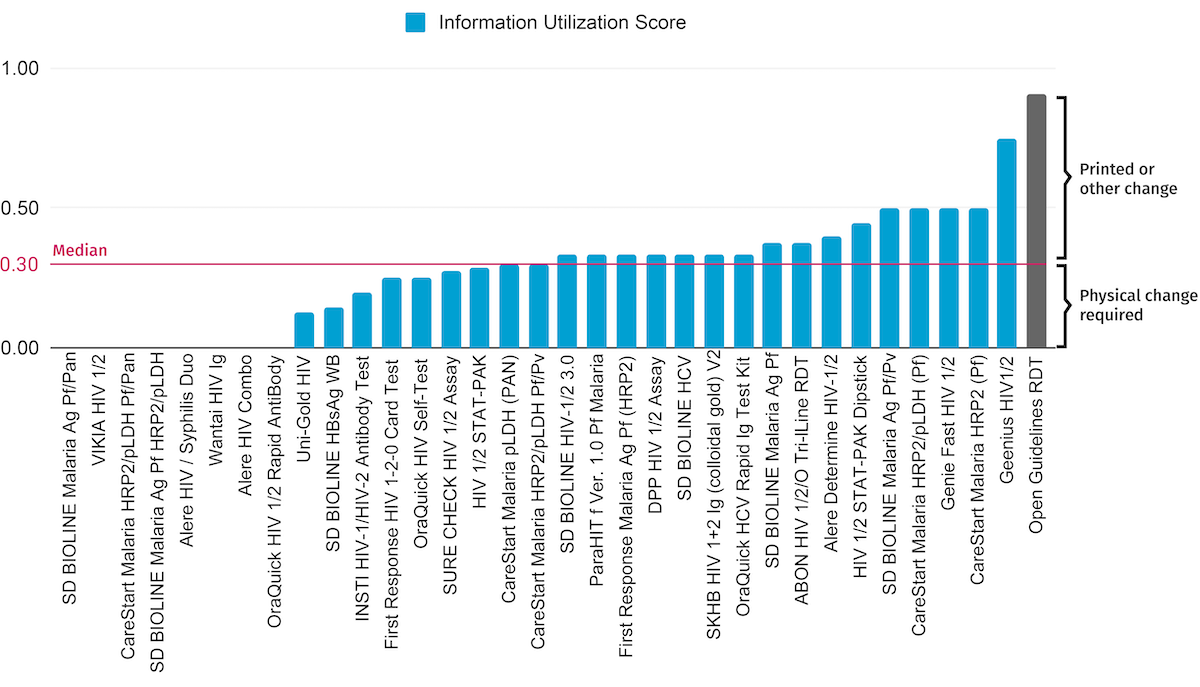
Information utilization index for WHO prequalified rapid diagnostic tests (RDTs). Scores were calculated for 33 WHO prequalified devices that had accessible information (blue, names listed below), as well as an RDT based on the RDT Open Guidelines (grey). The median information utilization score was 0.30 (magenta line), in contrast to 0.91, the score for an Open Guidelines RDT. 70% (top bracket) of the Open Guidelines RDT score can be attributed to non-physical changes, while the remaining 30% (bottom bracket) requires physical changes to the RDT.

The IUI—which provides an overview of how much information current diagnostics can capture and where there is room for improvement—for prequalified RDTs and an OG RDT had values ranging from 0 to 0.75 (mean 0.27; median 0.30, IQR 0.25) (Figure 4). The large bracket shows that 70% of this information usage score can be attributed to printed or other nonphysical changes, while the remaining 30% require physical changes to the RDT.

## Discussion

### RDT Open Guidelines

**Figure 5:**
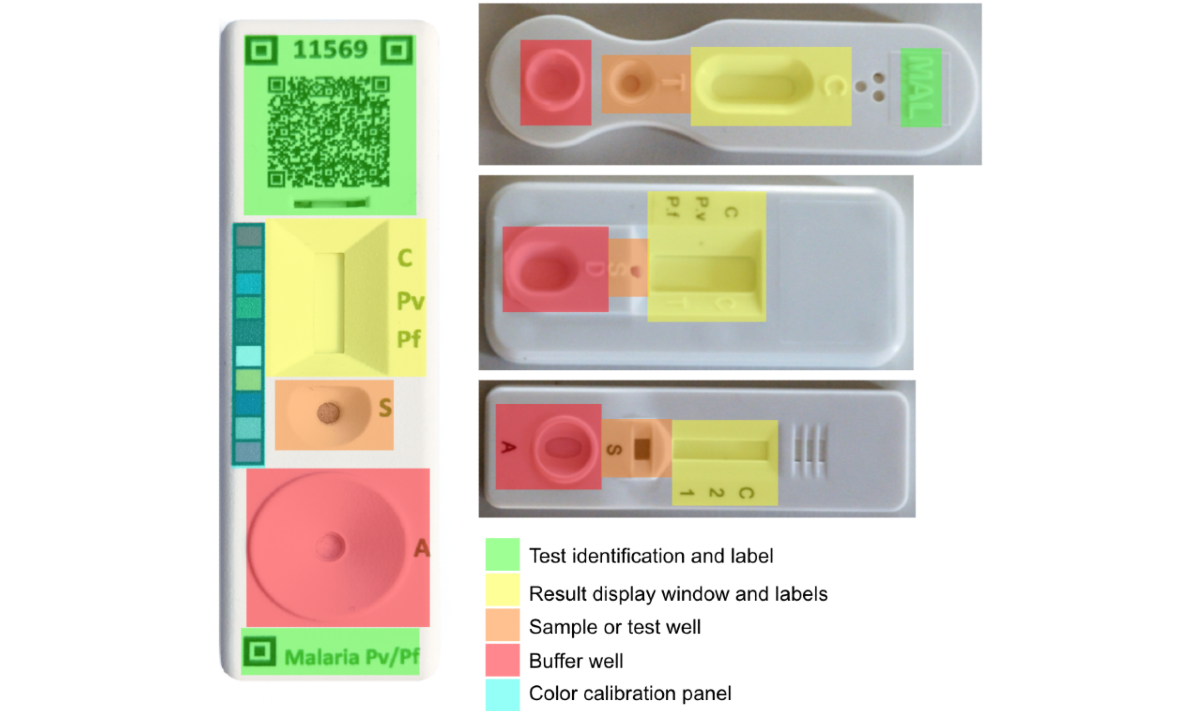
Unifying rapid diagnostic test functionalities based on formal guidelines. A rapid diagnostic test based on the rapid diagnostic test open guidelines (left) should have certain functional components, as indicated by the color-coded overlay. In contrast, 3 RDTs currently on the World Health Organization–prequalified list have only some of these components (right, with color-coded overlays).

Our RDT-OGs recommend the horizontal integration of RDT hardware through consistent physical modules, thereby enabling vertical integration of RDT software through consistent protocols linking supply chain, test choreography, and interpretation. In Figure 5, a reference example of RDT-OGs with colored overlays identifying the core modifications is shown. These include a 2D barcode to embed information needed for an app to identify, read, and interpret the RDT; fiducials as reference points to assist the camera and phone to quickly and accurately identify the RDT areas of interest and reference; and a color calibration panel to enable reliable colorimetric inference. In addition, 3 WHO-prequalified RDTs with overlays are shown to highlight current inconsistencies between tests (Figure 5).

In comparing the optimized IUI and design of the reference RDT-OGs to others, we observed both the heterogeneity and common structures across all RDTs. We have designed RDT-OGs to be useful whether adopting all recommendations, a subset of modules, or using existing cassettes linked to an RDT-OG–compatible software platform. Defining common data models and schemas provides an information architecture that would encapsulate data from any module combination that exists on the RDT. The RDT-OG data schema can be effectively encoded by the 2D barcode and easily drive the process forward via a reader app. The design and production aspects have proved feasible given the successful production of the prototype, and the field assessments, such as assessment of integration with epidemiological monitoring systems, are ongoing.

**Figure 6:**
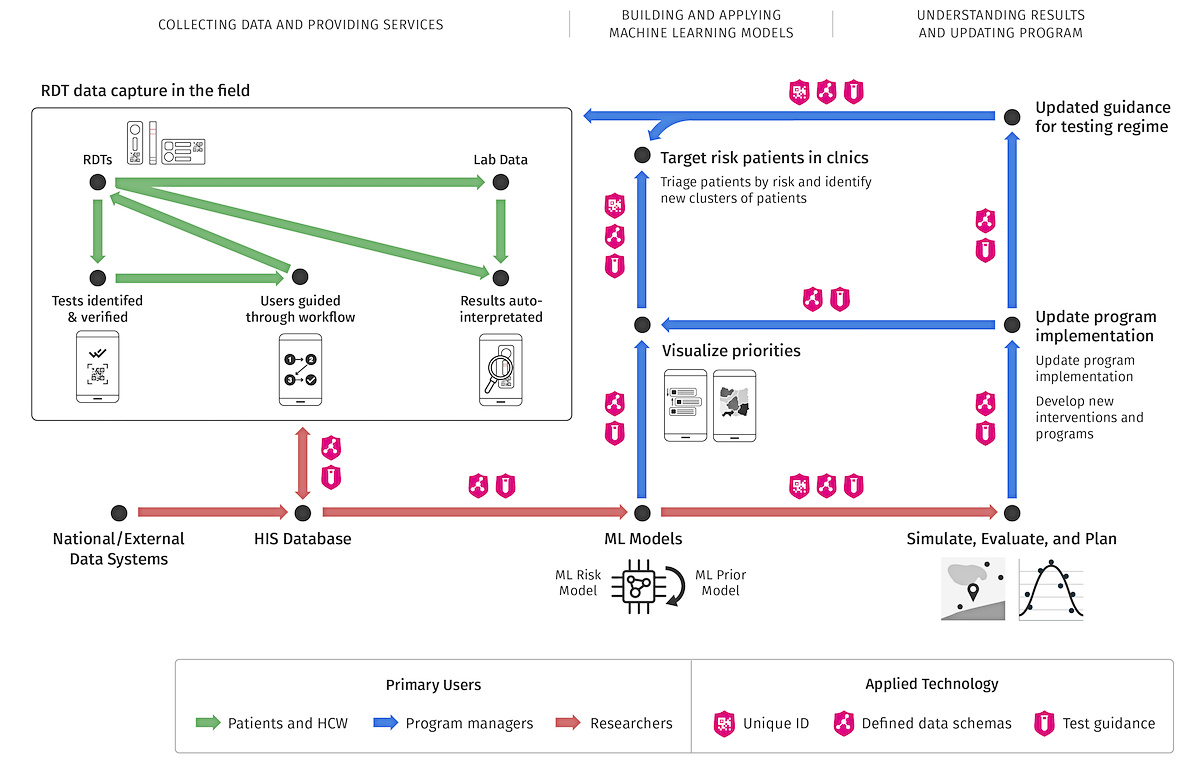
In a system that incorporates rapid diagnostic test open guidelines, data are captured and digitized data from rapid diagnostic tests using a smartphone app that is compatible with the rapid diagnostic test open guidelines. These data are transmitted to a health information system platform and integrated with health system data, laboratory data, and other relevant data, then used to build machine learning models that both feed upstream, to smartphone apps to model symptoms and to be used to better interpret results, as well as downstream, for monitoring. Planners, managers, and researchers can use the real-time data to decide on modifications to existing programs and plan new programs. The color of the lines identifies the primary participant in that portion of the workflow, and the badges depict where to apply the features of the rapid diagnostic test open guideline. ML: machine learning; RDT: rapid diagnostic tests.

**Figure 7:**
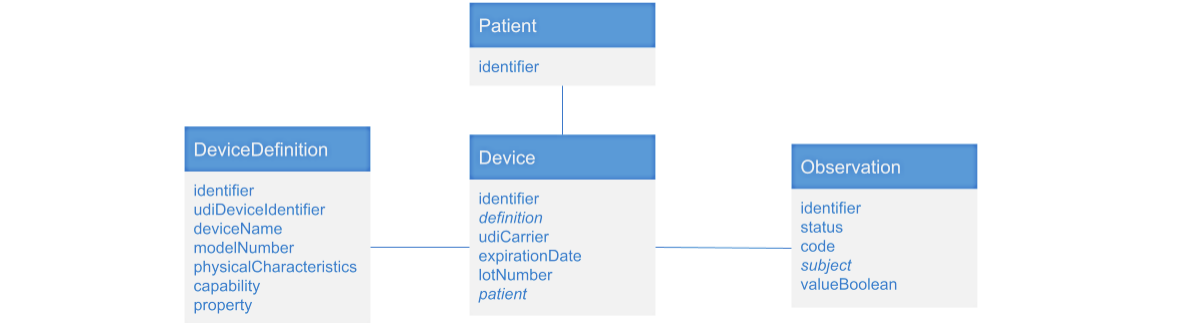
A Fast Healthcare Interoperability Resources–based workflow using the Device Definition resource for Open Guidelines based rapid diagnostic tests connected to Device, Observation, and Patient resources. Medical devices are defined using the DeviceDefinition resource (to specify their physical characteristics and links to external information systems). Each rapid diagnostic test used corresponds to a Device resource linked to the appropriate DeviceDefinition resource, as well as to Patient and Observation resources that store patient information and test results, respectively.

Creating a systematic way (Figure 6) to collect and aggregate structured RDT data allows the community to continuously monitor device performance, disease prevalence, and the relationship between demographic priors and diagnostic outcomes. This workflow, like the RDT-OG, is not tied to a particular diagnostic and is designed to accommodate both existing and emerging diagnostics. RDTs can increase their IUI by using a universal RDT-OG–compatible reader and data storage. New RDTs that become available in the market can further increase their IUI by using the hardware recommendations of the RDT-OGs (Figure 6). The workflow integrates automatic result interpretation modules using machine learning from image libraries or template-based approaches and can dynamically accommodate new RDTs through database-backed parameters defining rapid diagnostic test components and hyperparameters identifying the specific RDT (Figure 7). The rapidly growing number of COVID-19 serology and antigen-based RDTs show the critical role of dynamically supporting newly released RDTs [13,14].

To the best of our knowledge, this is the first paper to propose guidelines to harmonize the hardware, software, and data standard used to read and interpret RDTs.

### Diagnostics

#### Overview

It is useful to review the RDT-OG system with related hardware, software, and standard-based approaches to field diagnostics, and to note limitations and next steps to integrate the RDT-OGs into the digital health ecosystem.

#### Integrated Hardware-Based Field Diagnostics

Hardware-based field diagnostics require reusable equipment to function and connect to software systems. For example, the DekiReader is a portable device that guides users through a malaria RDT, reads, and automatically interprets test results; it is notable that its results are not significantly different from human readings [6,15]. Similarly, NutriPhone pairs a lateral flow cassette with a hardware device and app to guide users and process images of test results to measure vitamin B_12_ levels. It has not been tested at scale; however, in a sample of 12 participants, there was a correlation of 0.93 with the results from an immunoassay [16].

#### Software-Based Field Diagnostics

In contrast, software-based field diagnostics do not require additional hardware to function, while having accuracy comparable to human interpretations of RDTs or images of used tests [17]. Dell and Borriello [18] made use of the pre-existing Open Data Kit to read various cassette-based RDTs using only smartphones and 3D printable stands for consistent image capture. Similarly, Ozkan and Kayhan [19] developed an RDT holder that clips onto smartphones to improve image consistency and data interpretation. Demonstrating the utility beyond cassette-based RDT formats, Ra et al [20] combined a urine test strip with color calibration markers and a smartphone app to improve automated urinalysis accuracy across various lighting conditions. There are also proprietary RDT-reading platforms, including BBI Solutions’ Novarum Smartphone Reader and Abingdon Health’s AppDx Smartphone Reader, that integrate on-cassette QR codes but with limited information (such as RDT type) [21,22].

Though these approaches integrate modern software, their generalizability is limited by having been designed in the absence of guidelines that standardize their solutions, and therefore do not adhere to PPH axioms 2 and 3. Vashist et al [23] reviewed how smartphone-based health care apps and devices, including related medical, privacy, and data standards, remain fractured without guidelines or standards. A recent review [24] further extended the number of diagnostic devices and companies involved, and again concluded there is a lack of unification.

#### General Challenges in Field Diagnostics

Yager et al [25] describe the biomedical engineering community as historically focused on laboratory-based diagnostics and highlight the work needed to adapt tools for settings in low- and middle-income countries. Improvement in test instructions, health worker training, and performance monitoring all correlate with reduced preanalytical errors, improved test performance, and increased result reliability [7,26,27].

There is a growing consensus that point-of-care diagnostics and smartphones equipped with digital health solutions are converging and that this advancement may significantly expand self-managed care [28,29]. However, we currently lack digital health interventions with diagnostics linked to clinical care pathways and infectious disease surveillance systems [30], as well as solutions to the privacy and data stewardship challenges necessary for large-scale deployment [28]. Despite these challenges, researchers have outlined numerous promising future point-of-care linkages: handheld ultrasounds and software platforms with standardized databases integrating artificial intelligence [31], telecytology platforms for test-and-not-treat strategies [32], and accurate diagnoses of oral cancer using convolutional neural networks [33].

### Related Regulations and Standards

Existing standards for diagnostics encompass RDTs, including several related standards in health technology, medical devices, and precision medicine. For example, given that most rapid diagnostic tests have maximal temperature limits for storage and use, temperature exposure monitors such as those used on vaccine vials would be warranted based on performance degradation from heat exposure [34]. In addition, use of the Fast Healthcare Interoperability Resources (FHIR) [35] can define clinical data as a graph of well-defined fields and data types (Figure 7). A number of current digital lab data platforms and application programming interfaces already handle related diagnostic and laboratory information management with FHIR as a common standard [36].

Regulators, such as the US Food and Drug Administration, define pathways to classify novel medical devices, including communication-enabled RDTs, for which there are no similar existing devices [37]. Similarly, the Medical Device Communications Testing Project from the National Institute of Standards and Technology is relevant to any form of medical device communication and applicable to RDTs which communicate via radio frequency or electrochemical means [38]. These regulatory bodies also promote innovation (eg, the National Institute of Standards and Technology Text Retrieval Conference, where annual precision medicine competitions model the most effective treatments) exemplifying how communities can benefit from well-structured data [39].

### Limitations

This study has some limitations. First, the response rate from the survey was 40% (33/81), and the survey results may have benefited from additional feedback from a broader group. Nevertheless, there was strong thematic concordance between core responses from the survey and findings from the literature review. Second, after designing the open guidelines, we did not solicit additional feedback from the same and similar groups of persons who were contacted for the survey. This additional step would serve to validate the utility of the open guidelines. We note that our goal was to collect feedback from RDT producers and users of RDT-OGs. Third, we limited ourselves to information usage issues and solutions for cassette-based rapid tests and did not include the simpler dipstick type strips. However, the same concepts would apply and would need to be implemented in a way that is compatible with the lower space and cost profile of such tests [40]. Despite these caveats, the proposed RDT-OG approach is clearly applicable to the majority of RDTs currently deployed globally, and to those likely to be produced in the future as multiplex and complex tests become the norm.

### Conclusions and Policy Recommendations

In response to the goals and ambitions of the RDT community, we defined PPH axioms and derived RDT-OGs. The recommended modular foundation is designed to accelerate current RDT development, fieldwork, and successfully translate RDTs into effective field evaluations and deployments at scale. These guidelines thus confer functionality to diagnostic devices, the smartphone apps interpreting them, and the health information system analyzing them. For example, temperature sensors may be essential to assure proper storage and quality of some rapid diagnostic tests [34], and the modularity of open guidelines can accommodate this need. Although modifying supply chains may be infeasible in areas with rigid logistics or fixed asset costs, the RDT-OGs gives the community a pathway to extend the functionality of pre-existing field-based diagnostics through advances in machine learning, which do not require RDT modifications.

National and global policy makers have shown a willingness and ability to convene communities around guidelines that benefit RDT stakeholders; for example, the WHO prequalification of medicines program, FHIR, SNOMED, and LOINC. As the WHO, the Global Fund, Foundation for Innovative New Diagnostics, and others continue this work, there is ample opportunity to adopt formal guidelines around RDTs and their usage. For example, the WHO’s role in creating and promoting prequalified malaria RDTs has incentivized manufacturers to increase low-cost RDT production [41,42]. A similar approach to incentivize machine-readable RDT identifiers, and data schemas to interpret them, would likely address challenges currently faced.

Thus, by providing guidance for RDT hardware, software, and data interoperability, standards-setting organizations can transform RDTs into a formidable public health tool for disease prevention and treatment, in addition to diagnosis. These innovations can accelerate long-term disease control efforts, such as for malaria, which is responsible for 7.8% of the annual deaths of children under 5 years old (20,000 children worldwide [43]). Furthermore, these innovations can accelerate rapidly evolving disease control efforts, such as for COVID-19, where serological or antigen detection investigations face challenges in obtaining case information; these challenges are expected to further increase as testing efforts continue to scale up, and with transition from mitigation to containment [44]. Therefore, in both routine and emergency scenarios, adopting RDT-OGs would apply key advances in information technology to close the critical gap between diagnostics and public health interventions, and enable a new era of precision public health.

## Appendix

Multimedia Appendix 1 Survey instrument.

## Abbreviations

FHIR: Fast Healthcare Interoperability Resources
IUI: information utilization index
OG: open guideline
PPH: precision public health
RDT: rapid diagnostic test
WHO: World Health Organization
