# Supplementary material for: Transforming Rapid Diagnostic Tests for Precision Public Health: Open Guidelines for Manufacturers and Users
Source: JMIR Biomed Eng. 2022 Jul 29;7(2):e26800. doi: 10.2196/26800 (PMC11041428; doi:10.2196/26800)
Supplement: Multimedia Appendix 1 [file biomedeng_v7i2e26800_app1.pdf]

Ona Systems

Questionnaire for the feasibility and features needed for an open standard for RDT manufacturing for optimized machine reading on mobile devices. (version 3.0)

Subject areas are designated as G=general, U=user, M=manufacturer, I=informatics

How are you involved with RDTs? [G]:

a. Doctor or nurse
b. Community health worker
c. Client/user
d. Manufacturer
e. Seller
f. Researcher

Where are you from? [G]:

a. High income country
b. Low or middle income country

What is the main RDT domain you have experience with? [G]:

a. Infectious diseases
b. Maternal health
c. Substance abuse
d. Environmental testing

Assess the following statements based on this scale:
0 strongly disagree; 1 disagree; 2 neutral; 3 agree; 4 strongly agree; 5 not able to answer

1. Errors in RDT reliability are a serious problem [G].
2. Sources of reliability problems that are common and serious include [G]:
    a. Poor manufacturing standards
    b. Poor storage and shipping conditions
    c. Wrong biological specimen is used (e.g. urine instead of blood)
    d. Specimen is not obtained properly (e.g. too little blood)
    e. Specimen is not prepared properly (e.g. diluted too much)
    f. Proper reagents are not used
    g. Test timings and process flow is not executed correctly
    h. The test result is not read correctly
    i. The test result is read correctly but is not trusted for clinical decisions
3. Errors of health professionals in reading RDTs is a serious problem [GU].
4. Error of clients (e.g. ill persons or users) in reading RDTs is a serious problem [GU]
5. The requirement to use a hardware reader for RDTs is a barrier to use (example1: https://www.alere.com/en/home/product-details/alere-reader.html) [GUM]

6. The requirement to use a proprietary smart phone app to read RDTs is a barrier to use (example1: http://www.cellmic.com/content/rapid-test-readers example2: https://mobileassay.com/products/mobile-assay-reader) [GUM]
7. The requirement to use an open source free smart phone app to read RDTs is a barrier to use [GUM]
8. There is need for an open standard for producing RDTs that are optimized for reading and interpretation by an app on a smart phone [GUMI]
9. Reading an RDT with a smart phone app is useful for the following RDT layouts yielding a positive or negative test (i.e. non-quantitative) [GUMI]:
    a. Single test line with no control
    b. Single test line with a positive control
    c. Two sets of test lines with paired positive controls, and interpreted independently, i.e. pathogen A or pathogen B.
    d. Two sets of test lines with paired positive controls, and interpreted in an integrated way, i.e. 0,0 or 1,0 or 0,1 is no disease; 1,1 is disease.
    e. Three or more test lines with positive controls (independent or integrated)
10. Reading an RDT with a smartphone app is useful for the following RDT layouts yielding a signal interpreted quantitatively or semi-quantitatively [GUMI]:
    a. Single test line with no control
    b. Single test line with a positive control
    c. Two sets of test lines with paired positive controls, and interpreted independently, i.e. pathogen A or pathogen B.
    d. Two sets of test lines with paired positive controls, and interpreted in an integrated way, i.e. 0,0 or 1,0 or 0,1 is no disease; 1,1 is disease.
    e. Three or more test lines with positive controls (independent or integrated)
11. An open standard for manufacturing RDTs should include the following general domains for information that would be integrated into the test or its cassette independent of any packaging or wrapper [UMI]:
    a. Disease or condition being assessed (e.g. malaria, pregnancy)
    b. Manufacturing serial number of the specific test
    c. Counterfeit detection
    d. Expiration date of test
    e. Storage conditions (e.g. temperature or humidity)
    f. Other
12. Based on your knowledge or experience, the following characteristics for an RDT are likely to be important for readability using a mobile phone camera [GUM]:
    a. Overall shape of the cassette
    b. Cassette color
    c. Cassette material reflectance
    d. Depth of trough where test of result is visible
    e. Surface reflectance of surface of the test
    f. Transparent cover present over the test area
    g. Width of test or signal area
    h. Color of signal for test result
    i. Presence of a positive control

    j. Presence of a negative control
    k. Presence of colorimetric scale on test itself for colorimetric tests
    l. Other (should go to open listing text box)
13. The open standard for RDTs should extend beyond cassette manufacturing and include standardization of elements of test execution [GUM]:
14. The following elements of RDT standards for test execution are likely to be important [UM]:
    a. reagents being used
    b. sample preparation equipment
    c. Setting, e.g. temperature, lighting
    d. timings for sample application and test development
    e. Other
15. The open standard for RDTs should extend beyond cassette manufacturing and test execution, and include standardization of data concepts for test result coding and sharing [UMI]
16. An open standard for RDT manufacturing would be linked with a complementary standard of specific data that would need to be obtained from each RDT that was read. Defining these specific types if data would be important to support use of the RDTs manufactured to an open standard. In this context, please indicate you view on the importance of the following types of data [GUMI]:
    a. Condition being assessed
    b. Type of test (e.g. immunochromatographic, chemistry, colorimetric)
    c. Form factor
    d. Manufacturer
    e. Result (positive, negative)
    f. Patient symptoms crucial for interpretation of test result
    g. Ontologies for test interpretation, i.e. combinations of different data that define a condition (e.g. malaria clinical illness defined as current RDT positive and current fever)
    h. Geographic location of test
    i. Date and time of test
    j. Demographics of the person being assessed
17. RDTs optimized for smartphone reading will increase demand for widespread use of RDTs [GUM]
18. Requirements for RDT manufacturing to an open standard will create the following serious barrier for production [GUM]:
    a. Lack of manufacturing ability to create the tests that meet the standard
    b. Increase in costs
    c. Risk for proprietary information loss
    d. Other
19. Substantial advantages for manufacturers of RDTs that conform to open standard would be [GUM]:
    a. An immediate access to a market with users and clients who use the smartphone app
    b. The ability to know the performance of their product in different settings
    c. access to validated support and control features that should improve performance
    d. potentially accelerated path to testing and regulatory
    e. Other

20. Manufacturers would be willing to produce tests that conform to the open standard for the following reasons [GUM]:
    a. It is more profitable
    b. The use of the tests is more reliable
    c. It is required by law
    d. It is recommended by WHO or other standard setting organizations
21. An RDT that could only be read by a smartphone app (and not a human) would overcome potential human errors. (e.g. an RDT with a readout based on NFC/RFID) [GUM].
22. An RDT that could only be read by a smartphone app (and not a human) would be more trustworthy to the user [GUM].
23. Specific capabilities that would make a non-human readable RDT more trustworthy would be [GUM]:
    a. An app that gives an explanation of any QC/user error issues
    b. An app that integrates of population-level characteristics and priors (e.g. epidemic seasonality) for an accurate positive predictive value
    c. A digital report of the result that is sent to the user
    d. A written report that is printable by the user
24. Barriers to acceptability for non-human readable RDT's would be [GUM]:
    a. Increased cost
    b. The tests would more difficult to maintain quality in manufacturing
    c. The tests would be more difficult to assess for degradation
    d. Clinicians or users would not trust the app readout
    e. Clients would refuse to accept the results
    f. The tests would be more susceptible to fake tests and fake readers
    g. The RDT would require a phone or reader, and this would limit use
    h. Regulatory approval would be more difficult
    i. Other
25. When reading an RDT on a phone app, the following issues will affect validity [GUM]:
    a. different cameras on different phones
    b. dirt, scratches, or a clear cover on the camera lens
    c. automated image processing done by the operating system before the app reads the phone
    d. other
26. To overcome the issues of phone to phone variability, and different lighting conditions, these methods would be useful for calibration [UMI]:
    a. standard color stripes printed on the cassette
    b. standard color stripes on a chart placed next to the cassette when being read by the phone
    c. standard color stripes that appear on the test surface when the test is used
    d. other

27. Please list any other persons or groups who you think should receive this questionnaire [GUMI]?
